# Suicide Attempt by Glass Shard Ingestion: A Case Report

**DOI:** 10.7759/cureus.26312

**Published:** 2022-06-24

**Authors:** José R Alves, Luis F Spengler, Paola F David de Souza, Rodrigo O Lanza de Miranda

**Affiliations:** 1 Department of Surgery, Federal University of Santa Catarina, Florianópolis, BRA; 2 Department of Surgery, Florianópolis Hospital, Florianópolis, BRA; 3 Department of Sugery, Florianópolis Hospital, Florianópolis, BRA

**Keywords:** gastro-intestinal surgery, general trauma surgery, emergency medicine and trauma, suicide attempts, gi endoscopy

## Abstract

A 42-year-old man with schizophrenia and human immunodeficiency syndrome swallowed several glass shards in an attempted suicide. Two days later, he was admitted to the ER of the Florianópolis Hospital with a complaint of upper abdominal pain. The patient showed normal vital signs on physical examination; there was tenderness of palpation of the epigastrium. The investigation on admission included hemogram, CXR, abdominal X-ray, and upper GI scope. Radiographs showed several radiopaque image fragments in the stomach, as well as in the small and large intestines. Hemogram showed normal results. Upper gastrointestinal endoscopy found no signs of esophagogastroduodenal perforation; several glass shards were removed from the patient's stomach. The patient remained in the ward for four days and underwent continuous vital signs monitoring, serial physical examinations, hematimetric control, and daily imaging tests. He showed normal vital signs and progressive improvement of abdominal pain during hospitalization, although hematochezia episodes took place during defecation. The patient no longer complained of abdominal pain on the fifth hospitalization day; the complete removal of the glass fragments was confirmed through imaging examinations, and the patient was transferred to a specialized hospital in order to better treat his psychiatric condition.

## Introduction

Schizophrenia stands out among the mental disorders most commonly associated with suicide and suicide attempts, mainly when it is associated with depression, anxiety, and alcoholism [1]. Several reasons (pica or even hyalophagia, suicide attempt, among others) may lead people to ingest glass shards and/or fragments that may result in esophagogastroduodenal and intestinal perforations, infectious complications (infection/abscess), as well as upper and/or lower gastrointestinal bleeding [2-6]. The current study presents a case report concerning an adult patient who attempted to kill himself by ingesting several glass shards. Although the patient had small lower gastrointestinal bleeding (with no hemodynamic abnormality), he didn't need surgical intervention to eliminate all glass fragments.

## Case presentation

A 42-year-old man with schizophrenia (auditory hallucinations) and human immunodeficiency syndrome (not using specific antiretroviral medication) broke two glass cups and ingested several shards in an attempt to kill himself after he had an argument with some family members. Two days later, he sought emergency care, complaining of upper abdominal pain. The patient showed normal vital signs at the time of physical examination; however, he felt pain on deep epigastric palpation.

**Figure 1:**
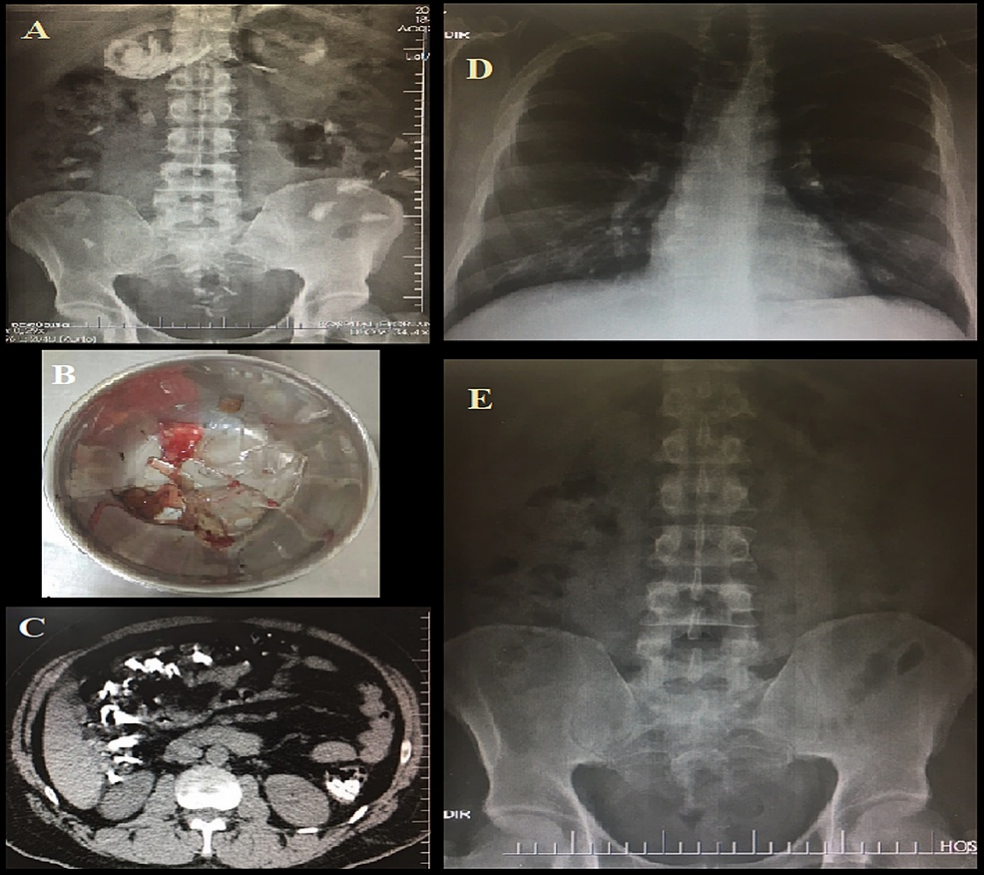
Radiographic and upper gastrointestinal endoscopy findings Panel A: radiograph of an orthostatic abdomen (first hospitalization day) showing several radiopaque fragments (glass) inside the stomach and in the intestine. Panel B: metal container holding several glass shards that were removed from the patient's stomach through upper gastrointestinal endoscopy. Panel C: total abdomen tomography (without intravenous contrast) performed for control purposes (second hospitalization day) showing glass shards inside the intestine, although with no signs of pneumoperitoneum. Panels D-E: radiological control performed on the last hospitalization day showing the complete elimination of the glass shards and lack of pathological intracavitary air signs.

The patient was subjected to serum tests, chest and abdomen radiographs, as well as upper gastrointestinal endoscopy at the time he was hospitalized. The radiographs showed several radiopaque image fragments distributed in the stomach, as well as in the small and large intestines; however, there was no sign of pneumoperitoneum (Figure 1A). The C-reactive protein was 13.4 mg/dl (reference value up to 5 mg/dl); the coagulogram showed normal results; and hemogram showed normal hematimetry, leukocytes of 4,500/mm^3^ (lymphocytes of 1,035/mm^3^) and normal platelets. No signs of esophagogastroduodenal perforation were found in the upper gastrointestinal endoscopy, although small clots were scattered in the gastric mucosa, and several glass shards were removed from the patient's stomach (Figure 1B).

The physician in charge adopted conservative conduct - performing clinical observation with serial physical exams three times/day or in case of clinical deterioration; performing hematimetric and radiological control on a daily basis; keeping the patient fasted using crystalloid solutions, and prescribing 40 mg of omeprazole/day based on the tests results, normal vital signs and on the abdominal exam presenting no signs of pneumoperitoneum/peritonitis. A helical abdominal tomography (without intravenous contrast) was also performed on the second hospitalization day in order to check the absence of pneumoperitoneum or other complications that could not have been previously identified in radiographs (Figure 1C). At that time, the patient stopped defecating; however, he kept on expelling flatus and showed persistent epigastralgia, although it was less intense. At the beginning of the fourth hospitalization day, the patient was able to defecate after he was instructed to intensify deambulation. However, according to the patient and the nursing team, although his feces showed normal color, there was a small amount of blood around them. Thus, a thorough rectal examination was performed, and it confirmed the absence of blood in the rectal ampulla. The hematimetric controls kept on showing no significant decrease throughout hospitalization (hemoglobin variation of 12.4-14.5 g/dl, hematocrit variation of 37.8-44.8%). The leukogram remained in the normal range (from 4,500 to 6,100/m^3^), whereas the C-reactive protein progressively reduced until its normalization on the last hospitalization day (fifth day). The patient remained calm and tried no further suicide attempts. In addition, he was hemodynamically normal throughout the treatment, and an abdominal exam showed progressive epigastralgia reduction until he became asymptomatic. A light laxative diet (using probiotics) was introduced on the fourth hospitalization day and was well accepted by the patient. Radiographs performed on the fifth hospitalization day confirmed complete glass fragment elimination and showed no signs of perforation (Figures 1D-E). Since the hospital had no psychiatric service, the patient was transferred to another institution in order to better treat his psychiatric condition.

Written informed consent was obtained from the patient's guardian for publication of this case report and any accompanying images.

## Discussion

The epigastralgia presented by the patient since hospitalization may be related to gastric mucosa lesions/inflammation caused by the glass fragments, and although it lacks literature embasement, the use of omeprazole might have a role in the patient's clinical improvement. In addition, the hematochezia was probably justified by the presence of small lesions in the colonic mucosa during the glass fragments elimination. However, it did not cause great concern because the patient remained hemodynamically normal, and the hematimetric control showed no changes. It is assumed that the patient's digestive tract had no perforation because he swallowed large glass fragments with blunt edges [5].

Performing a colonoscopy to remove the remaining glass fragments during the patient's evolution was considered but not performed because this exam requires intracolonic air injection that could increase the peristalsis and distend the intestine, thus potentially increasing the risk of perforations.

## Conclusions

The ingestion of glass shards and/or fragments is a high-risk situation that can cause complications that require immediate surgical treatment. Thus, it is recommended to continuously monitor (clinical, laboratory, and image) the patient until the presence of complications or until the elimination of all glass shards/fragments. Surgery is indicated in case of complications (perforation, bleeding with persistent hemodynamic abnormality, and infection/abscess).
